# A Novel Method for Quality Evaluation of *Gardeniae fructus* Praeparatus during Heat Processing Based on Sensory Characteristics and Chemical Compositions

**DOI:** 10.3390/molecules27113369

**Published:** 2022-05-24

**Authors:** Yinghao Zheng, Yun Wang, Qing Zhang, Weihong Liu, Kai Li, Mengyu Xia, Zhe Jia, Cun Zhang

**Affiliations:** 1 Institute of Chinese Materia Medica, China Academy of Chinese Medical Sciences, Beijing 100700, China; yinghao200888@163.com (Y.Z.); 15210014020@163.com (Y.W.); 17638706927@163.com (Q.Z.); xiamengyu1215@163.com (M.X.); jiaz127@163.com (Z.J.); 2 College of Pharmacy, Henan University of Chinese Medicine, Zhengzhou 450046, China; cpulikai@163.com; 3 The First Affiliated Hospital of Henan University of Traditional Chinese Medicine, Zhengzhou 450046, China; lwhc@163.com

**Keywords:** *Gardeniae fructus* Praeparatus, quality assessment, electronic eye, electronic nose, electronic tongue, correlation analysis

## Abstract

The intrinsic chemical components and sensory characteristics of *Gardeniae fructus* Praeparatus (GFP) directly reflect its quality and subsequently, affect its clinical curative effect. However, there is little research on the correlation between the appearance traits and chemical compositions of GFP during heat processing. In this study, the major components of five typical processed decoction pieces of GFP were determined. With the deepening of processing, the contents of geniposidic acid and 5-HMF gradually increased, while the contents of deacetyl-asperulosidic acid methyl ester, gardenoside, and two pigments declined. Moreover, the electronic eye, electronic tongue, and electronic nose were applied to quantify GFP’s sensory properties. It was found that the chroma values showed a downward trend during the processing of GFP. The results of odor showed that ammonia, alkenes, hydrogen, and aromatic compounds were the material base for aroma characteristics. Complex bitterness in GF was more obvious than that in other GFP processed products. Furthermore, one mathematical model was established to evaluate the correlation between the sensory characteristics and chemical composition of GFP during five different stages. A cluster analysis and neural network analysis contributed to recognizing the processing stage of GFP. This study provided an alternative method for the exterior and interior correlation-based quality evaluation of herbs.

## 1. Introduction

Chinese medicine processing, a preparation technology (used to make Chinese medicine decoction pieces more suitable under the guidance of Chinese medicine theory) is an important step before the clinical application of traditional Chinese medicine (TCM) [1]. The inherited chemical components and sensory characteristics including color, taste, and odor could reflect the quality of TCM and then affect the clinical curative effect [2]. Meanwhile, the empirical theory of quality assessment from the character identification of TCM would be interpreted objectively based on the correlational analyses between appearance characteristics and active ingredients [3]. A growing number of researchers focus on the correlation between sensory features and chemical components, but research on single appearance signature are relatively common; for example, main active ingredients and quantitative color values were important bases for the quality evaluation of *Gentianae macrophyllae radix* with different drying methods [4]. The quality of *Corni Fructus* was closely related to its color, so it can be identified and graded by exterior color [5]. The color values and chemical components served as the criteria for quality evaluation and processing of the end-point determination of rhubarb charcoal [6]. A good correlation between index composition and powder color was observed during the microwave processing of *Cibotium baronetz* which can be used to monitor processing [7]. Odor characteristics that exhibited a significant correlation with chlorogenic acid provided a reliable basis for the quality control of *Lonicera japonica* [8]. Quantifying taste information was regarded as a meaningful approach that controlled the quality of herbal product sage lozenges [9]. To investigate the research further, comprehensive morphological characteristics combining internal ingredients have attracted attention. A novel quality evaluation method with HPLC, an electronic eye, and an electronic nose was applied to assess the quality of magnolia bark [10]. It was possible to distinguish Hawthorn and its processed products based on compounds, color, odor, and flavor [11]. Overall, external-interior correlation analysis plays an important role in the processing of TCM.

*Gardeniae fructus* (GF) is the dried and ripe fruit of *Gardenia jasminoides* ellis of the Rubiaceae family; the fruitcan be processed into *Gardeniae fructus* Praeparatus (GFP) to change the medicinal properties and meet the different clinical requirements of syndrome differentiation in TCM. Currently, some efforts have been made to study the changes in the processing of GFP. A strategy by UPLC-ESI-QTOF and a multivariate statistical analysis found that some chemical changes existed between GF and GFP, showing downward trends of geniposide, genipin-1-*O*-gentiobioside, 6a-hydroxygeniposide, jasminoside B, crocin, and mannitol, while mussaenosidic acid increased [12]. The study with the aid of a UPLC characteristic chromatogram and color value demonstrated that there were significant differences in crocins as well as chroma values between GFP and other processed products [13]. However, equally important qualities such as the odor and flavor of GFP are ignored, let alone their relationship to intrinsic ingredients. Thus, it is necessary to establish an objective and accurate quality evaluation system for GFP. 

The previous study by our group has preliminarily identified five processed decoction pieces of GFP that reflected the different and typical processing stages, namely pre-processed pieces (GF), middle-processed pieces (GFP-M), near-processed pieces (GFP-N), the right processed product (GFP) and far-processed pieces (GFP-F) [14]. In this study, a comprehensive evaluation of five processed decoction pieces of GFP was carried out by UPLC, an electronic eye, electronic nose, and electronic tongue. More importantly, data fusion from multi-source information was performed to explore the relationships between intrinsic ingredients and apparent characteristics by correlation analysis. This study could provide supervision for the processing of GFP based on cluster analysis and artificial neural network analysis. 

## 2. Results

### 2.1. Determination of Content in Five GFP Processed Decoction Pieces

**Figure 1:**
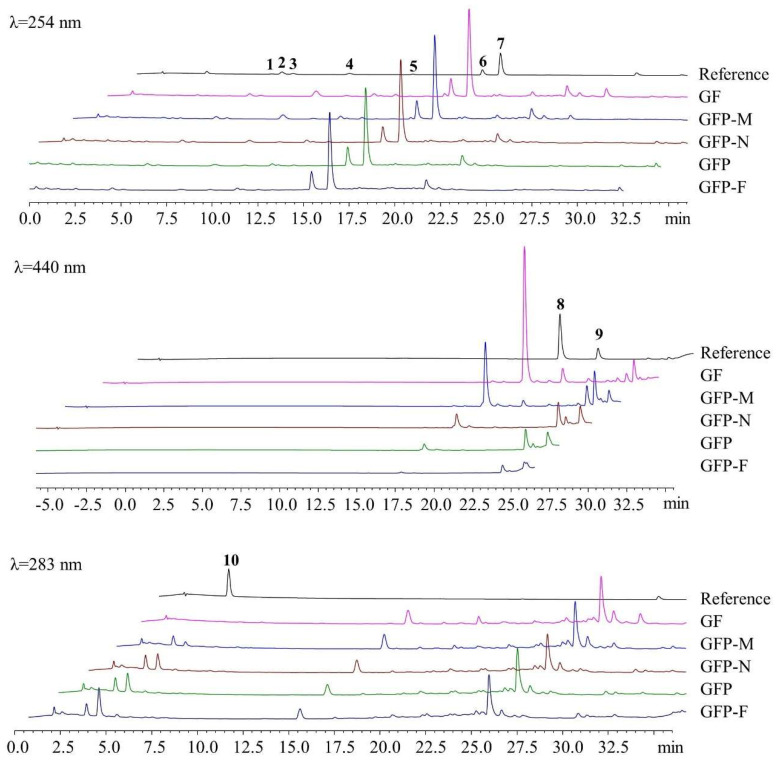
UPLC chromatogram of five GFP processed decoction pieces. 1—geniposidic acid; 2—shanzhiside; 3—deacetyl-asperulosidic acid methyl ester; 4—gardenoside; 5—scandosidemethyl ester; 6—G1; 7—G2; 8—C-I; 9—C-II; 10—5-HMF.

**Figure 2:**
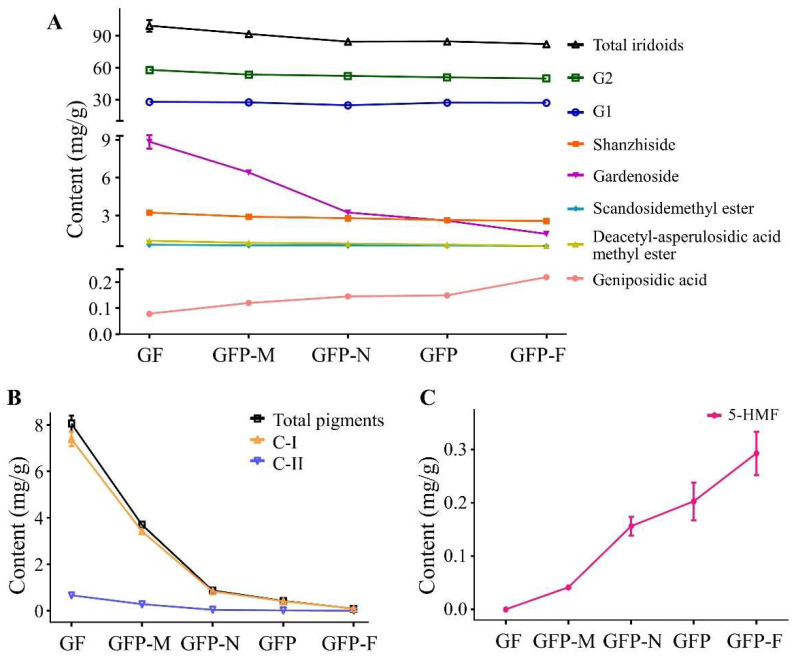
The changed contents of 10 compounds of GF (pre-processed pieces), GFP-M (middle-processed pieces), GFP-N (near-processed pieces), GFP (the right processed product) and GFP-F (far-processed pieces). (**A**) Iridoids; (**B**) Pigments; (**C**) 5-HMF.

We determined the contents of the main effective compositions of GF, GFP-M, GFP-N, GFP, and GFP-F by UPLC. The chromatograms are shown in Figure 1. Seven iridoid glycosides were identified at 254 nm, including geniposidic acid, shanzhiside, deacetyl-asperulosidic acid methyl ester, scandosidemethyl ester, gardenoside, genipin-1-O-gentiobioside (G1), and geniposide (G2). Detection of crocin-I (C-I) and crocin-II (C-II) at 440 nm showed that their response values decreased with the deepening of the processing degree and even the C-II peak was not obvious in the GFP-F sample. Meanwhile, 5-HMF was identified at 283 nm. Notably, it could not be detected in GF but its response value gradually increased in the deeper processed samples. The quantitative results are displayed in Table S1 and Figure 2. As shown in Figure 2A, deacetyl-asperulosidic acid methyl ester in GFP-F significantly decreased as compared to GF (*p* < 0.05); the contents of gardenoside in GFP-N, GFP, and GFP-F were significantly different from that of GF (*p* < 0.05 or *p* < 0.01). The content of shanzhiside, scandosidemethyl ester, gardenoside, and G2 were on a downward trend, but there was no significant difference. The content of geniposidic acid increased gradually during the heating process (*p* < 0.001). Overall, there was a falling-off in the contents of total iridoids with no great difference. Pigments such as C-I and C-II decreased sharply with the deepening of processing and they were significantly reduced in four GFP processed decoction pieces (*p* < 0.001 compared to GF). Even C-II could not be detected in GFP-F (Figure 2B). The contents of the total pigments were reduced significantly (*p* < 0.05). In addition, as seen in Figure 2C, 5-HMF was formed during the heating processing and its content gradually increased (*p* < 0.05 or *p* < 0.01).

### 2.2. Color Analysis of Five GFP Processed Decoction Pieces

**Figure 3:**
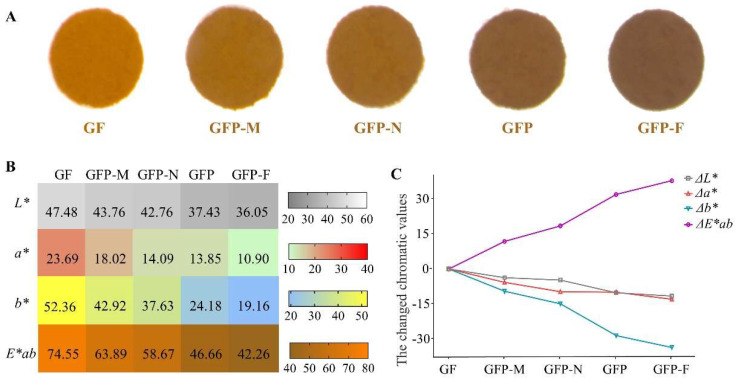
The result of color analysis of GF (pre−processed pieces), GFP−M (middle−processed pieces), GFP−N (near−processed pieces), GFP (the right processed product), and GFP−F (far−processed pieces). (**A**) Intuitive colors. (**B**) The values in the heatmap represent the corresponding L*, a*, b*, and E*ab values of each decoction piece. The L* value from large to small indicates luminance from light to dark. The a* value from large to small indicates color from red to green. The b* value from large to small indicates color from yellow to blue. The E*ab values from large to small mean color change from GF to GFP-F. (**C**) The values of ΔL*, Δa*, and Δb* represent the changed chroma values of L*, a*, and b* compared to the original value of GF, respectively. ΔE*ab represents color difference values.

In the heating process of GFP the apparent color of the samples showed intuitive changes that the color gradually deepened, changing from yellow orange to yellowish-brown and finally turning tan (Figure 3A). Chroma values for each sample are shown in Figure 3B. The L* value with the range of 0~100 indicated colors from black to white. It is found that the L* of decoction pieces during processing went down with the time extended. In the case of a higher a* value, the color that neared red conversely neared green. Compared with the GF in zero point, the a* value of samples with deeper processing decreased continuously. When the b* value became larger the color turned yellow, otherwise, it turned blue. The b* value decreased significantly during the processing of GFP. E*ab showed a downward trend indicating that the color of GFP processed products changed from red and yellow to brown. As seen in Figure 3C, ΔL*, Δa*, and Δb* all decreased, among which Δb* largely declined with the largest slope. Meanwhile, ΔE*ab with the ranges from 0 to 37.37 showed that the color difference of the samples during continuous processing increased gradually compared with the initial decoction piece. 

### 2.3. Olfactory Analysis of Five GFP Processed Decoction Pieces

**Figure 4:**
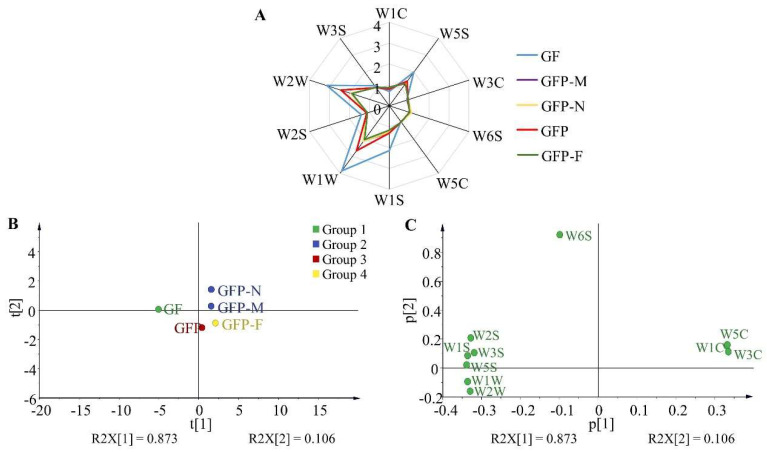
The odor information of five GFP processed decoction pieces. (**A**) Radar map of response value. (**B**) PCA analysis of electronic nose data. (**C**) Loadings analysis of electronic nose data.

The electronic nose was applied for a quantitative analysis of the aromatic features of five GFP processed decoction pieces, and the signal responses of sensor arrays informed that a stable signal output at 70 s was identified as the odor value (Figure S1). The odor composition of five GFP processed decoction pieces was basically the same but differed in the response intensity of each odor (Table S2 and Figure 4A). The response values of W1C, W3C, and W5C from GF were lower than that of the other four processed GFP decoction pieces, while the response values of W5S, W6S, W1S, W1W, W2S, W2W, W3S from GF were higher than other products. The scores scatter plot of the principal component analysis (PCA) is displayed in Figure 4B. With the first component accounting for 87.80% of the variables and the second principal component for 10.20%, the accumulative variance contribution of the two principal components accounted for 98.00%. Five GFP processed decoction pieces were distributed in different regions without overlap. Among them, GFP-M and GFP-N which were intermediate between GF and GFP could be regarded as a group as they were assigned to close positions. From this, the electronic nose could identify different stages of the GFP processed process according to the odor variation, that is, GF (before processing), GFP-M and GFP-N (processing was not up to the moderate point), GFP (processing was in a moderate point), and GFP-F (processing is far away than a moderate point). According to the loadings analysis results, the parameters of W1C, W3C, and W5C in the first principal component and W6S in the second principal component had higher scores. These sensors could recognize ammonia, alkenes, hydrogen, and aromatic compounds, which might be the important substance bases resulting in the odor difference of GFP processed decoction pieces (Figure 4C). 

### 2.4. Gustatory Analysis of Five GFP Processed Decoction Pieces

**Figure 5:**
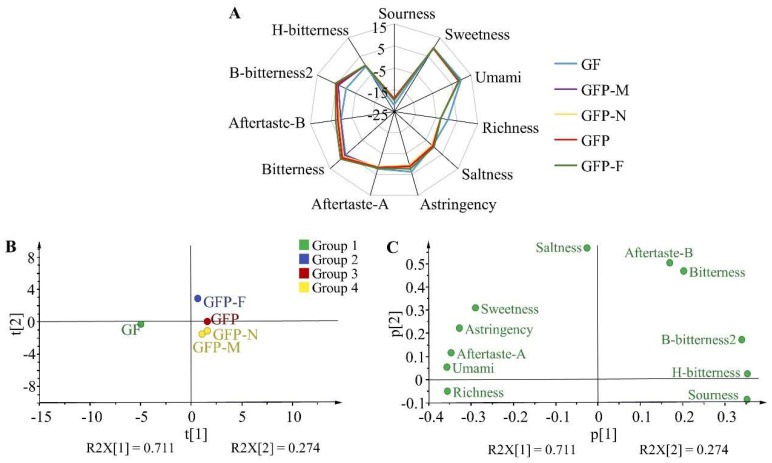
The taste information of five GFP processed decoction pieces. (**A**) Radar map of response value. (**B**) PCA analysis of electronic tongue data. (**C**) Loadings analysis of electronic tongue data.

The taste–response value of the reference solution was bench-marked so the initial values of sourness and saltiness were identified as −13 and −6, respectively, and the initial values of other tastes were 0. The quantitative taste results of five GFP processed decoction pieces are shown in Table S3 and Figure 5A. The taste difference between GF and another four GFP processed decoction pieces was significant and there was little difference in the four GFP products. As shown, five GFP processed decoction pieces did not contain sourness and H-bitterness as their responses were lower than the initial value. The sweetness could be recognized in all samples with almost the same response value and the response of umami in GF was slightly higher than that of the other four types of GFP. The richness of the umami aftertaste derived from GF but might be lost during processing. The saltiness, astringency, and aftertaste-A were higher in GF, decreased successively in GFP-M and GFP-N, then gradually increased in GFP and GFP-F. Significantly, the response values of bitter taste, acidic bitter taste, and alkaline bitter taste increased with the processing degree, indicating that a large number of bitter ingredients could be produced during the processing of GFP. A PCA analysis showed that the first and second principal components encompassed 71.10% and 27.40% of the contributing rate which covered the main information of five GFP processed decoction pieces with a 98.8% cumulative contribution of variance (Figure 5B). Each decoction piece was distributed in different regions in the PCA scores scatter plot. It is worth noting that GFP-M was close to GFP-N in that they had similar flavor features. Overall, the electronic tongue could figure out the different processed products of GFP according to the smell messages. Concretely, GFP-M and GFP-N were classified into one group while GF, GFP and GFP-F were the other three groups. A loadings analysis was performed to determine the flavors that differentiated the five GFP processed decoction pieces. As shown in Figure 5C, bitterness, aftertaste-B, and B-bitterness2 contributed significantly to the first and second principal components; saltiness had the highest loading parameters in the second principal component, followed by sweetness, astringency, and aftertaste-A which contributed to the second component. Thus, seven flavors including bitterness, aftertaste-B, B-bitterness2, saltiness, sweetness, astringency, and aftertaste-A were regarded as primary indicators for distinguishing the difference in flavor profile among GFP processed decoction pieces.

### 2.5. Integration Analysis of External Characteristics and Internal Components

#### 2.5.1. Correlation Analysis

**Figure 6:**
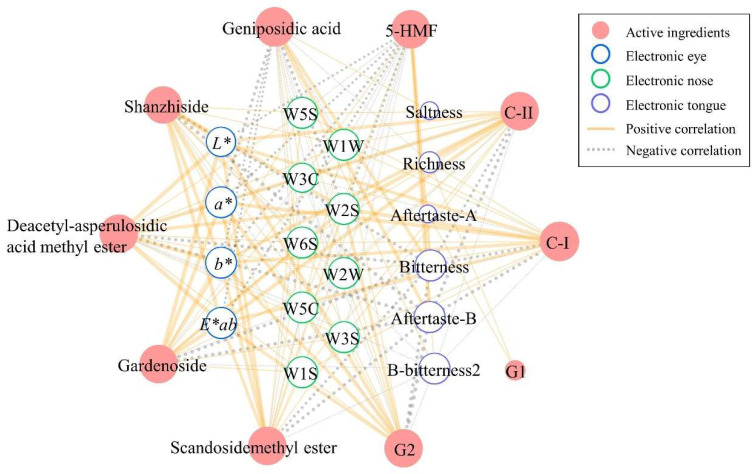
The relationship network presents significant correlations between the components and the indexes of external features. The a* indicates color from red to green, b* represents color from yellow to blue, and E*ab means total chromatic aberration.

A correlation analysis was performed between the main active ingredients and the quantified sensory indexes of five GFP processed decoction pieces. The first consideration was to assess the normal distribution of the variables and homogeneity of variance. As a result, geniposidic acid, b*, E*ab, sweetness, saltiness, and bitterness simultaneously satisfied the two requirements (Table S4) which were performed with a Pearson correlation analysis. Other factors were analyzed by a Spearman correlation test. As shown in Table S5, except for G1, the other nine components had different degrees of correlation with the appearance indexes. The chroma values including L*, a*, b*, and E*ab were positively correlated with shanzhiside, deacetyl-asperulosidic acid methyl ester, gardenoside, scandosidemethyl ester, G2, C-I, and C-II, and were negatively correlated with geniposidic acid and 5-HMF. As for the relationship between aroma and components, W5S, W6S, W1S, W1W, W2S, W2W, and W3S had a significant positive association with shanzhiside, deacetyl-asperulosidic acid methyl ester, gardenoside, scandosidemethyl ester, G2, C-I, and C-II, while they were inversely correlated with geniposidic acid and 5-HMF. W3C and W5C were associated with a positive value of geniposidic acid and 5-HMF. W3C was negatively correlated with deacetyl-asperulosidic acid methyl ester, gardenoside, scandosidemethyl ester, G2, C-I, and C-II, and we also found that there was a significant negative correlation between W5C and these six compounds and shanzhiside. In the correlation analysis of flavor and composition, richness was significantly and negatively correlated with geniposidic acid but positively correlated with G1 and C-I. Saltiness showed a strong correlation with geniposidic acid. There was a strong correlation between aftertaste-A and G1. Further, a strong relationship with *p* < 0.01 appeared between three kinds of bitter taste (bitterness, aftertaste-B, and B-bitterness2) and nine main ingredients (geniposidic acid, shanzhiside, deacetyl-asperulosidic acid methyl ester, gardenoside, scandosidemethyl ester, G2, C-I, C-II, and 5-HMF). Specifically, the correlation coefficient between B-bitterness2 and nine components was the highest, regardless of positive or negative correlation, compared to bitterness and aftertaste-B. The relationships between the main active ingredients that were significantly related to the indicators of color, odor, and taste are visualized in Figure 6. As shown, the more connections to other nodes, the larger the node. The edges connecting two nodes represent the correlation between them, and thicker edges mean stronger connections. Meanwhile, solid orange lines indicate a positive correlation between the two factors, while the dashed gray ones present a negative correlation. In the whole network, four color indexes, nine sensor arrays, and six taste indexes were correlated with internal substance components.

#### 2.5.2. Cluster Heatmap Analysis

**Figure 7:**
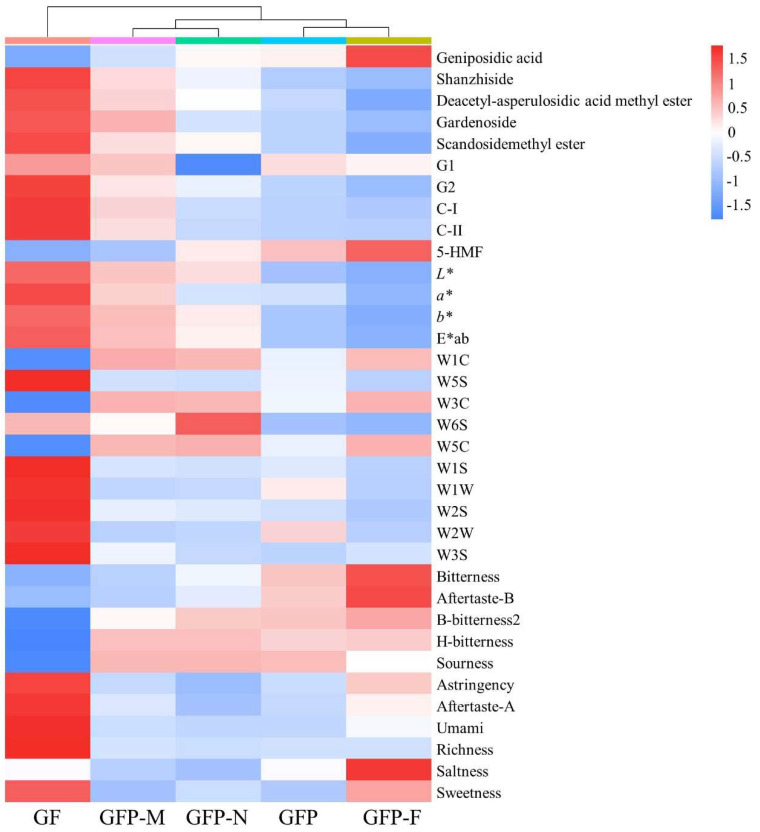
Cluster heatmap for estimating the processing stage of GFP. The scale bar of the heatmap represents Z-score normalized.

Data fusion of external characteristics and internal components was successfully applied to estimate the processing stage of GFP. The cluster heatmap was plotted for a comprehensive analysis of the indexes, including ingredients, color, flavor, and taste (Figure 7). It was obvious that GF was significantly different from the other four pieces and was regarded as a separate group. GFP-M and GFP-N were combined into a group because of the weaker difference between their internal and external features. Further, GFP came close to GFP-F, so they were considered as one class. Overall, gradual changes occurred when GF processed decoction pieces was produced in a more deeply processed GFP, whether with internal components or external features.

#### 2.5.3. Artificial Neural Network Analysis

**Figure 8:**
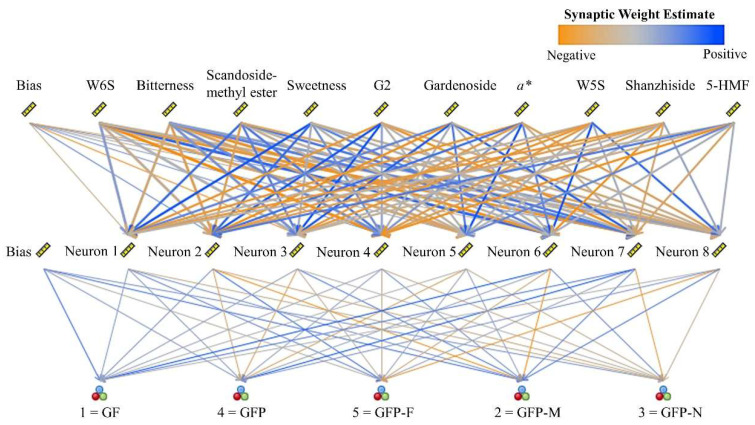
Artificial neural network for classification of five GFP processed decoction pieces.

To classify GFP processed samples more accurately we constructed an artificial neural network as the machine-learning classifier. Thirty-five nodes representing sensory characteristics and chemical compositions were fed into the input layers; eight units were in the hidden layer and five GFP processed decoction pieces were used as outputs. The different importance of the variables for the neural network model is displayed in Figure S2. The network structure is presented in Figure 8 and focuses on the top ten most important predictors, such as W6S, bitterness, scandosidemethyl ester, sweetness, G2, gardenoside, a*, W5S, shanzhiside, and 5-HMF. The connection lines in the diagram are colored according to an estimate of the synaptic weight, and greater line width corresponds to greater importance. The neural network had a high prediction rate of 100% (Figure S3), suggesting the validity of classification.

## 3. Discussion

As intelligent bionic systems mature, the electronic eye, electronic nose, and electronic tongue combined with analytical instruments are applied for the quality discrimination of TCM processed products. For instance, Xu et al. [15] successfully applied intelligent sensory technology and chromatographic analysis technology in the identification of *Semen arecae* and its processed products. The researchers measured the color values of crude and processed *Leonuri fructus* using the intelligent sensor technology of a colorimeter to assess their quality [16]. The analysis of the sensory index of Chinese herbal decoction pieces followed by a correlation analysis of the appearance and internal chemical composition of the decoction pieces can better reveal the correlation between the appearance and internal quality of the decoction pieces. It is an inevitable trend to establish a scientific and practical quality evaluation system of TCM decoction pieces for reflecting the characteristics of TCM. Here, an attempt in the present work has been made to explore the relationships between the internal components and external characteristics of GFP by Pearson and Spearman correlations. Satisfyingly, color characters had a good correlation with the main ingredients, including geniposidic acid, shanzhiside, deacetyl-asperulosidic acid methyl ester, gardenoside, scandosidemethyl ester, G2, C-I C-II, and 5-HMF. It is reported that the higher the contents of total crocins and C-I, the redder GF [17]. With the deepening of GFP processing, the contents of C-I and C-II dropped significantly due to the breaking of bonds during the heating process. As a result, a* value indicated the gradual decline in red property, identifying a positive correlation with C-I and C-II. As reported in the literature, the Maillard reaction occurred during heat processing when the product 5-HMF was formed [18]. The content of 5-HMF that was produced by the Maillard reaction rose with increased heating time and temperature [19]. Thus, the content of 5-HMF continued to grow during the heat processing of GFP. In addition, 5-HMF played a role in browning [20], and this work found the intuitive phenomena associated with it. 5-HMF increased and L* value decreased successively in deeper processed products, indicating that the 5-HMF might be related to a color change from bright to dark. Nine electronic nose sensors (W2S W5S, W3C, W6S, W5C, W1S, W1W, W2S, W2W, and W3S) had correlations with geniposidic acid, shanzhiside, deacetyl-asperulosidic acid methyl ester, gardenoside, scandosidemethyl ester, G2, C-I, C-II, and 5-HMF in varying degrees, indicating that odor characteristics were closely related to the quality of GFP. A burnt smell could be produced during the processing of GFP which may be related to the aromatic components that were identified by these sensors. Five GFP processed decoction pieces had a complex taste of bitterness, an acidic–bitter aftertaste, and alkalescent bitterness. The bitterness of GF was mainly derived from geniposide [21]. However, deeper GFP processed products with a lower content of geniposide showed stronger bitterness, suggesting that other bitter substances would be produced during the processing of GFP. Most likely, the Maillard reaction promoted the formation of various products with significant bitterness, such as 5-HMF [22]. Therefore, the tastes of bitterness, acidic–bitter aftertaste, and alkalescent bitterness negatively correlated to most iridoid glycosides, as well as showing high positive correlations with 5-HMF. Together, these results of correlation analysis might facilitate our understanding of the scientific connotation of the morphological identification of GFP. It is an important feature of this study to establish a holistic quality evaluation system of GFP by combining quantitative sensory attributes with an internal material basis. 

Importantly, these obtained results enable judgment on the processing stage of GFP. It was satisfying to determine whether electronic nose or electronic tongue technology could be used for monitoring the processing of GFP, separately. Five GFP processed decoction pieces were independent of each other, among which GFP-M was close to GFP-N as they had a certain common flavor and taste. Further, the overall cluster analysis of data fusion, including ingredients, color, aroma, and taste proposed the division of stages during the processing of GFP. Not only were previous phases approaching GFP (namely GFP-M and GFP-N) classified as one group, but GFP and GFP-F were also closely associated. The artificial neural network was better at classifying complex data with efficiency and accuracy [23] which showed an ideal classification performance on GFP processed decoction pieces with a prediction rate of 100%. In aggregate, the integration strategy based on the exterior and interior characteristics is expected to provide a reference for the overall quality evaluation of other decoction pieces and monitoring dynamic processes.

## 4. Materials and Methods

### 4.1. Materials and Chemicals

The typical processed decoction pieces of GFP were identified in the previous report [14]. Briefly, an electromagnetic stir-frying machine (CYJ 900, Beijing Hualin Ruikong Technology Co., Ltd., Beijng, China) was used to prepare GFP processed decoction pieces and GFP. The processed decoction pieces were sampled continuously every 0.5 or 1 min for 15 min in the processing of GFP, among which the sample at 12.5 min was judged as qualified GFP by an experienced pharmacist. As recorded in the *Chinese Pharmacopoeia* (2020 edition), the surface was brown or black, and the inner surface and seed surface of pericarp was yellowish brown or tan [24]. Subsequently, all samples were clustered based on their component contents and chroma values in order to screen five representative decoction pieces spanning from initiation to far exceeding endpoint: GF (0 min), GFP-M (6 min), GFP-N (10 min), GFP (12.5 min), and GFP-F (15 min). Geniposidic acid, shanzhiside, and C-II were purchased from Chengdu Chroma-Biotechnology Co., Ltd. (Chengdu, China). C-I and 5-HMF were bought from Chengdu Must Bio-Technology Co., Ltd. (Chengdu, China). Scandosidemethyl ester was offered by Chengdu Lemetian Medicine Technology Co., Ltd. (Chengdu, China). The purities of these commercially available reference substances were above 98% as determined by HPLC analysis. Gardenoside, deacetyl-asperulosidic acid methyl ester, G1, and G2 were prepared by our laboratory with purity over 98% by HPLC analysis. Methanol, formic acid, absolute alcohol, and concentrated hydrochloric acid were provided by Sinopharm Chemical Reagent Co., Ltd. (Shanghai, China). Potassium chloride (KCl), potassium hydroxide (KOH), and DL-tartaric acid were acquired from Tianjin Kemiou Chemical Reagent Co., Ltd. (Tianjin, China). Silver chloride (AgCl) was obtained from Shanghai Macklin Biochemical Co., Ltd. (Shanghai, China).

### 4.2. Preparation of Samples and Reference Substance

The sample solution was prepared following the reported protocol [14]. Briefly, GF, GFP-M, GFP-N, GFP, and GFP-F were crushed into powder using a pulverizer for 2 min and passed through a 40 μm mesh sieve, then 0.5 g of each sample was accurately weighed and placed into a 50 mL conical flask. After adding 25 mL of 50% methanol, the samples were extracted by ultrasound for 30 min (Kun Shan Ultrasonic Instruments Co., Ltd., Jiangsu, China). The sample solution was cooled down to room temperature and weighed again, and then the lost weight was restored to the original weight with 50% methanol. The continuous filtrate was collected, followed by filtering with 0.22 μm of organic membranes. Samples were prepared in triplicate for UPLC analysis. 

The mixed reference substance was dissolved with 50% methanol which contained 5.75 μg/mL of geniposidic acid; 64.00 μg/mL of shanzhiside; 15.90 μg/mL of deacetyl-asperulosidic acid methyl ester; 31.80 μg/mL of gardenoside; 8.80 μg/mL of scandosidemethyl ester; 157.00 μg/mL of G1; 279.00 μg/mL of G2; 45.50 μg/mL of C-I; 10.10 μg/mL of C-II; and 4.95 μg/mL of 5-HMF.

### 4.3. UPLC Analysis

Five typical processed decoction pieces of GFP and the mixed reference substances were determined according to the reported method. [14] Briefly, UPLC (Nexera XR LC-20AD XR, Shimadzu, Kyoto, Japan) was operated with a Waters Acquity UPLC BEH C_18_ column (2.1 mm × 100 mm, 1.7 μm) at the temperature of 35 °C. The gradient elution with 0.5% formic acid (A) and methanol (B) was performed as follows: 0–6 min, 6% B; 6–11 min, 6–14% B; 11–19 min, 14–40% B; 19–24 min, 40~45% B; 24–29 min, 45–65% B; 29–30 min, 65–100% B; 30–36 min, 100% B. The flow rate was set as 1.0 mL/min, and the injection volume was 1 μL. The samples were detected at multiple wavelengths of 254 nm, 283 nm, and 440 nm.

### 4.4. Electronic Eye Analysis

Five kinds of powder from GFP processed decoction pieces were put into a dish, making the surface flat. First, an electronic vision analyzer (VA400, Alpha M.O.S, Toulouse, France) equipped with a CMOS lens needed to be calibrated with the color card, and then it was used to gather color information from the samples. The chroma values were converted into the parameters of CIELAB color space such as L* (Luminosity); a* (red/green); and b* (blue/yellow). E*ab (total color value) was calculated in the following formula: E*ab = (L*^2^ + a*^2^ + b*^2^)^1/2^, while ΔE*ab = [(L* − *L*_0_*)^2^ + (a* − *a*_0_*)^2^ + (b* − *b*_0_*)^2^]^1/2^, among which *L*_0_, *a*_0_, and *b*_0_ were determined based on GF.

### 4.5. Electronic Nose Analysis

The aroma of five GFP processed decoction pieces was discriminated by the electronic nose (PEN3, Airsense Analytics, Schwerin, Germany). Five samples were put into a headspace vial and sealed with plastic wrap, respectively. A needle injection probe was inserted into the sealed samples to acquire odor characteristics by direct headspace aspiration. The self-cleaning time of the sensors was 100 s and the sample preparation time was 5 s. The injection flow was 400 mL/min with a sampling time of 80 s. At 70 s, the sensor signal value was stable, so the data of this time were considered as the output value. The electronic nose had ten sensor arrays each responding to different sensitive substances. Specifically, W1C was sensitive to aromatic compounds; W5S recognized nitrogen oxides; W3C presented ammonia and aromatic compounds; W6S detected hydrogen; W5C responded to alkenes and aromatic compounds; W1S identified methane; W1W reflected sulfides compounds; W2S was related to alcohols and partially aromatic compounds; W2W indicated aromatic compounds and organic sulfides; and W3S corresponded to alkenes. The measured data were imported into Simca 14.1 software for a PCA and loadings analysis. The main idea of PCA was to reduce the dimensionality of data, transferring the original n-dimensional features to k-dimensional features (k < n) with little loss of whole-data information [25]. The PCA analysis was exploited in order to identify the differences or similarities between samples in an unsupervised mode to judge the degree of GFP processing. The loadings analysis would evaluate the influence of the variables on the components based on the distance from the variable to the origin [26], and it was used to identify better indexes that distinguished differences between the samples.

### 4.6. Electronic Tongue Analysis

An electronic tongue (TS-5000Z, Insent Company, Atsugi-Shi, Japan) containing multiple taste sensors simulates a flavor-recognition system which can distinguish bitterness, aftertaste-B, B-bitterness2, H-bitterness, astringency, aftertaste-A, umami, richness, sweetness, sourness, and saltiness. Five sample solutions were prepared by adding 3 g of sample to 100 mL of water, followed by an ultrasound for 10 min at 37 °C. The cleaning liquid for the positive electrode was prepared in a mixture with a total volume of 1000 mL consisting of 7.46 g KCl, 0.56 g KOH, and 300 mL absolute alcohol. As for the cleaning liquid of negative electrodes, mix 300 mL of anhydrous ethanol with 500 mL of deionized water, then add 8.3 mL of concentrated hydrochloric acid, and finally replenish to 1000 mL. Next, 0.18 g DL-tartaric acid and 8.946 g KCl were dissolved with 4000 mL pure water to prepare the reference fluid.

After washing in positive and negative electrode cleaning solutions for 90 s and the reference solution for 120 s twice, the sensor returned to zero at the equilibrium position for 30 s. The sample was tested for 30 s, outputting the first taste value. The sensor was cleaned and immediately inserted into a new reference solution to test the aftertaste for 30 s. Each sample was tested four times among which the first cycle was removed and the next three cycles were valid. The average data were retained as the test result. Similarly, the sweetness was tested for five cycles, retaining the data of the middle three times. Furthermore, the response values of the odor characteristics were analyzed by PCA and loadings analysis for preliminary identification of the different processed samples and important variables.

### 4.7. Multi-Source Data Integration

The multi-source information from internal components and external characteristics in terms of color, smell, and taste were integrated. These results were imported into SPSS 26 software for correlation analysis. Data were examined for normal distribution via the Shapiro-Wilk test and homogeneity of variance using the Levene test. Data that simultaneously satisfied both the normality and homogeneity of variance were analyzed using Pearson’s coefficient correlation, whereas variables were performed by Spearman’s correlation. When *p* < 0.05, a significant correlation was affirmed. Moreover, the relationship among correlational indicators (geniposidic acid, shanzhiside, deacetyl-asperulosidic acid methyl ester, gardenoside, scandosidemethyl ester, G1, G2, C-I, C-II, 5-HMF, L*, a*, b*, E*ab, W5S, W3C, W6S, W5C, W1S, W1W, W2S, W2W, and W3S, saltiness richness, aftertaste-A, bitterness, aftertaste-B, and B-bitterness2) was visualized in network form by Cytoscape 3.7.0 software (http://www.cytoscape.org, accessed on 23 April 2022). A cluster heatmap was performed on a free online platform (http://www.bioinformatics.com.cn, accessed on 23 April 2022) to judge the processing degree of GFP. The values of all indicators were normalized with a Z-score standardization method. By calculating each row of raw data into the normalized Z-score one by one, the data of different dimensions were normalized to the same dimension which helped to demonstrate the distribution of data. The calculation formula was as follows: Z-score = (raw value−mean)/standard deviation [27]. Then, a cluster analysis was conducted using Euclidean distance for two samples and a complete linkage method for two clusters. A neural network model with multilayer perceptron (MLP) was established by SPSS Modeler 1.0.0.430 software which consisted of an input layer, a hidden layer with eight nodes, and an output layer. The original datasets including five GFP processed decoction pieces in triplicate were split into a 70% training set and a 30% test set.

### 4.8. Statistical Analysis

The variance among groups was calculated using SPSS 26 software. The statistical significance of GF and other processed GFP products was assessed by one-way analysis of variance (ANOVA). An LSD test was used when the variance was homogeneous while Dunnett’s T3 test was used when the variance was non-homogeneous. A *p* < 0.05 was considered to be significant.

## 5. Conclusions

In summary, here, UPLC combined with intelligent sensory technologies including the electronic eye, electronic nose and electronic tongue were applied to analyze five representative GFP decoction pieces, followed by an integrated analysis of data from these instruments which was considered as a novel strategy for the quality evaluation of GFP and its processed products. Based on this, ten active ingredients were key indexes to evaluate the quality of GFP processed products and had definite correlations with digital chroma values. Aromatic compounds might be relevant to the burnt odor that occurs during heat processing. Bitterness, acidic–bitter aftertaste, and alkalescent bitterness were defining flavor features of GFP. Further, various processed GFP decoction pieces could be better identified by cluster analysis and artificial neural network analysis. Hence, the proposed method based on sensory characteristics and chemical compositions owns the advantages of objective evaluation, fast and simple operation, and is expected to succeed in assessing the quality and monitoring procedures of GFP during processing.

## Data Availability

The data presented in this study are available in Supplementary Material.

## Supplementary Materials

The following supporting information can be downloaded at: https://www.mdpi.com/article/10.3390/molecules27113369/s1, Table S1: The contents of 10 compounds among 5 processed products of GFP. Table S2: The response values of sensors of the electronic nose among five processed products of GFP. Table S3: The response values of sensors of the electronic tongue among five processed products of GFP. Table S4: Results of the normal distribution test of variables and homogeneity of variance. Table S5: Correlation between the components and the sensory characteristics (color, odor, and taste). Figure S1: Response curve of sensors of an electronic nose. Figure S2: Variable importance of neural network model. Figure S3: Overall percentage correction of the neural network model.

## Sample Availability

Samples of the compounds are available from the authors.
