# Supplementary material for: A Novel Method for Quality Evaluation of Gardeniae fructus Praeparatus during Heat Processing Based on Sensory Characteristics and Chemical Compositions
Source: Molecules. 2022 May 24;27(11):3369. doi: 10.3390/molecules27113369 (PMC9182132; doi:10.3390/molecules27113369)
Supplement: Supplementary file 1 [file molecules-27-03369-s001.zip › molecules-1718813-supplementary.pdf]

A Novel Method for Quality Evaluation of Gardeniae Fructus Praeparatus during Heat Processing Based on Sensory Characteristics and Chemical Compositions

Yinghao Zheng[1†], Yun Wang[1†], Qing Zhang[1,2], Weihong Liu[3], Kai Li[2], Mengyu Xia[1,2], Zhe Jia[1], Cun Zhang[1,2,#]

[1]Institute of Chinese Materia Medica, China Academy of Chinese Medical Sciences, Beijing 100700, China;

[2]College of Pharmacy, Henan University of Chinese Medicine, Zhengzhou 450046, China;

[3]The First Affiliated Hospital of Henan University of Traditional Chinese Medicine, Zhengzhou 450046, China

supplementary

Table S1. The contents of 10 compounds among 5 processing products of GFP (mg/g, n=3).

Compounds	GF	GFP-M	GFP-N	GFP	GFP-F
Geniposidic acid	0.08±0.01	0.12±0.00***	0.15 ±0.00***	0.15±0.00***	0.22±0.00***
Shanzhiside	3.24±0.19	2.91±0.02	2.79±0.01	2.64±0.02	2.56±0.05
Deacetyl-asperulosidic acid methyl ester	1.01±0.06	0.84±0.01	0.79±0.01	0.71±0.00	0.60±0.01*
Gardenoside	8.83 ±0.53	6.41±0.07	3.24±0.05*	2.59±0.02**	1.55±0.03**
Scandosidemethyl ester	0.69±0.03	0.65±0.00	0.64±0.01	0.62±0.00	0.60±0.00
G1	27.73±1.60	27.30±0.23	24.57±0.83	27.03±0.15	26.80±0.73
G2	57.71±3.15	53.32±0.40	52.18±0.49	50.79±0.28	49.71±0.84
Total iridoids	99.29±5.56	91.55±0.42	84.35±1.00	84.51±0.14	82.04±1.66
C-I	7.40±0.31	3.43±0.05**	0.83±0.01**	0.40±0.01**	0.08±0.00**
C-II	0.66±0.03	0.28±0.00**	0.04±0.00**	0.02±0.00**	-
Total pigments	8.06±0.35*	3.71±0.06*	0.87±0.01*	0.42±0.01*	0.09±0.00*
5-HMF	-	0.04±0.00**	0.16±0.02*	0.20±0.04*	0.29±0.04*

Note: The significance difference is indicated as * $p < 0.05$, ** $p < 0.01$, *** $p < 0.001$ compared to GF.

Table S2. The response values of sensors of electronic nose among 5 processed products of GFP (n=3).

Sensors	GF	GFP-M	GFP-N	GFP	GFP-F
W1C	0.68±0.00	0.87±0.00	0.86±0.00	0.80±0.00	0.86±0.00
W5S	1.99±0.00	1.38±0.00	1.37±0.00	1.46±0.00	1.31±0.00
W3C	0.88±0.00	0.96±0.00	0.96±0.00	0.93±0.00	0.96±0.00
W6S	1.07±0.00	1.05±0.00	1.10±0.00	1.02±0.00	1.01±0.00
W5C	0.94±0.00	0.97±0.00	0.97±0.00	0.97±0.00	0.98±0.00
W1S	2.16±0.00	1.29±0.01	1.26±0.01	1.32±0.00	1.17±0.00
W1W	3.86±0.01	2.09±0.00	2.11±0.00	2.67±0.00	2.00±0.01
W2S	1.41±0.00	1.15±0.01	1.15±0.01	1.13±0.00	1.09±0.00
W2W	3.14±0.00	1.89±0.00	1.93±0.00	2.43±0.00	1.87±0.01
W3S	1.19±0.00	1.09±0.00	1.07±0.00	1.06±0.00	1.07±0.00

Table S3. The response values of sensors of electronic tongue among 5 processed products of GFP (n=3).

Sensors	GF	GFP-M	GFP-N	GFP	GFP-F
Sourness	-22.06±0.08	-19.17±0.01	-19.21±0.03	-19.24±0.02	-19.92±0.03
Sweetness	9.44±0.05	9.28±0.08	9.31±0.07	9.29±0.06	9.40±0.05
Umami	9.84±0.01	8.71±0.02	8.67±0.02	8.66±0.02	8.92±0.02
Richness	0.80±0.07	-2.63±0.03	-2.71±0.02	-2.69±0.01	-2.64±0.01
Saltness	-0.85±0.68	-1.14±0.24	-1.23±0.27	-0.84±0.27	-0.05±0.29
Astringency	3.89±0.26	1.27±0.11	0.79±0.12	1.32±0.09	2.51±0.10
Aftertaste-A	2.58±0.02	1.87±0.02	1.68±0.01	1.79±0.00	2.04±0.02
Bitterness	5.35±0.33	5.90±0.06	6.57±0.10	7.30±0.11	8.47±0.12
Aftertaste-B	0.27±0.04	0.59±0.01	1.05±0.02	1.86±0.06	3.10±0.03
B-bitterness2	-0.04±0.12	4.02±1.04	4.90±0.65	4.99±0.32	5.57±0.72
H-bitterness	-0.58±0.04	0.00±0.03	0.00±0.02	-0.04±0.03	-0.03±0.01

Table S4. Results of normal distribution test of variables and homogeneity of variance.

	Normal distribution test		Homogeneity of variance	
	Significance	Note	Significance	Note
**Geniposidic acid**	**0.094**	**Normal distribution**	**0.081**	**Homogeneous variance**
Shanzhiside	0.074	Normal distribution	0.002	Non-homogeneous variance
Deacetyl-asperulosidic acid methyl ester	0.390	Normal distribution	0.001	Non-homogeneous variance
Gardenoside	0.021	Non-normal distribution	0.001	Non-homogeneous variance
Scandosidemethyl ester	0.162	Normal distribution	0.005	Non-homogeneous variance
G1	0.241	Normal distribution	0.009	Non-homogeneous variance
G2	0.015	Non-normal distribution	0.002	Non-homogeneous variance
CI	0.001	Non-normal distribution	0.011	Non-homogeneous variance
CII	0.001	Non-normal distribution	0.010	Non-homogeneous variance
HMF	0.111	Normal distribution	0.003	Non-homogeneous variance
*L**	0.036	Non-normal distribution	0.558	Homogeneous variance
*a**	0.031	Non-normal distribution	0.022	Non-homogeneous variance
b*	**0.056**	**Normal distribution**	**0.334**	**Homogeneous variance**
E*ab	**0.067**	**Normal distribution**	**0.702**	**Homogeneous variance**
W1C	0.000	Non-normal distribution	0.052	Homogeneous variance
W5S	0.000	Non-normal distribution	0.098	Homogeneous variance
W3C	0.000	Non-normal distribution	0.361	Homogeneous variance
W6S	0.031	Non-normal distribution	0.171	Homogeneous variance
W5C	0.000	Non-normal distribution	0.008	Non-homogeneous variance
W1S	0.000	Non-normal distribution	0.090	Homogeneous variance
W1W	0.000	Non-normal distribution	0.537	Homogeneous variance
W2S	0.000	Non-normal distribution	0.218	Homogeneous variance
W2W	0.000	Non-normal distribution	0.175	Homogeneous variance
W3S	0.000	Non-normal distribution	0.504	Homogeneous variance
Sourness	0.000	Non-normal distribution	0.020	Non-homogeneous variance
**Sweetness**	**0.839**	**Normal distribution**	**0.832**	**Homogeneous variance**
Umami	0.000	Non-normal distribution	0.554	Homogeneous variance
Richness	0.000	Non-normal distribution	0.010	Non-homogeneous variance
**Saltness**	**0.513**	**Normal distribution**	**0.419**	**Homogeneous variance**
Astringency	0.023	Non-normal distribution	0.237	Homogeneous variance
Aftertaste-A	0.004	Non-normal distribution	0.020	Non-homogeneous variance
**Bitterness**	**0.362**	**Normal distribution**	**0.187**	**Homogeneous variance**
Aftertaste-B	0.023	Non-normal distribution	0.225	Homogeneous variance
B-bitterness2	0.003	Non-normal distribution	0.218	Homogeneous variance
H-bitterness	0.000	Non-normal distribution	0.106	Homogeneous variance

Table S5. Correlation between the components and the sensory characteristics (color, odor, and taste).

	**Indexes**	**Geniposidic acid**	**Shanzhiside**	**Deacetyl-asperulosidic acid methyl ester**	**Gardenoside**	**Scandosidemet hyl ester**	**G1**	**G2**	**C-I**	**C-II**	**5-HMF**
Color	*L**	-0.950**	0.968**	0.982**	0.986**	0.946**	0.339	0.961**	0.954**	0.964**	-0.929**
	*a**	-0.957**	0.971**	0.979**	0.968**	0.964**	0.350	0.968**	0.968**	0.979**	-0.929**
	*b**	-0.917**	0.961**	0.979**	0.968**	0.954**	0.354	0.957**	0.957**	0.957**	-0.943**
	*E*ab*	-0.919**	0.961**	0.979**	0.979**	0.954**	0.350	0.957**	0.957**	0.968**	-0.932**
Odor	W1C	0.091	-0.107	-0.102	-0.104	-0.104	-0.141	-0.091	-0.098	-0.109	0.109
	W5S	-0.693**	0.671**	0.654**	0.668**	0.625*	0.321	0.671**	0.661**	0.671**	-0.700**
	W3C	0.514*	-0.507	-0.529*	-0.543*	-0.579*	-0.179	-0.525*	-0.546*	-0.554*	0.539*
	W6S	-0.683**	0.680**	0.683**	0.673**	0.755**	-0.288	0.673**	0.682**	0.671**	-0.624*
	W5C	0.580*	-0.574*	-0.603*	-0.631*	-0.581*	-0.317	-0.576*	-0.581*	-0.589*	0.610*
	W1S	-0.663**	0.695**	0.692**	0.685**	0.645**	0.399	0.685**	0.695**	0.706**	-0.695**
	W1W	-0.593*	0.593*	0.575*	0.582*	0.593*	0.007	0.589*	0.575*	0.586*	-0.593*
	W2S	-0.957**	0.968**	0.957**	0.950**	0.907**	0.207	0.950**	0.968**	0.979**	-0.936**
	W2W	-0.593*	0.589*	0.571*	0.586*	0.586*	0.007	0.579*	0.561*	0.571*	-0.589*
	W3S	-0.682**	0.680**	0.676**	0.673**	0.589*	0.453	0.667**	0.685**	0.685**	-0.642**
Taste	Sourness	0.029	-0.011	-0.018	-0.025	-0.052	-0.114	-0.027	-0.011	0.000	0.021
	Sweetness	-0.054	0.057	0.073	0.089	0.143	0.259	0.095	0.047	0.018	-0.113
	Umami	-0.361	0.328	0.341	0.350	0.325	0.486	0.352	0.328	0.317	-0.352
	Richness	-0.539*	0.483	0.510	0.501	0.465	0.612*	0.487	0.518*	0.500	-0.509
	Saltness	0.572*	-0.457	-0.450	-0.429	-0.432	0.421	-0.432	-0.479	-0.493	0.411
	Astringency	-0.150	0.171	0.175	0.161	0.104	0.500	0.154	0.182	0.193	-0.164
	Aftertaste-A	-0.279	0.300	0.295	0.305	0.212	0.606*	0.298	0.298	0.304	-0.313
	Bitterness	0.957**	-0.957**	-0.961**	-0.968**	-0.943**	-0.307	-0.968**	-0.954**	-0.943**	0.964**
	Aftertaste-B	0.960**	-0.981**	-0.974**	-0.969**	-0.960**	-0.331	-0.996**	-0.963**	-0.969**	0.962**
	B-bitterness2	0.794**	-0.839**	-0.819**	-0.792**	-0.753**	-0.261	-0.801**	-0.821**	-0.846**	0.742**
	H-bitterness	0.197	-0.190	-0.192	-0.215	-0.178	-0.368	-0.181	-0.115	-0.122	0.180

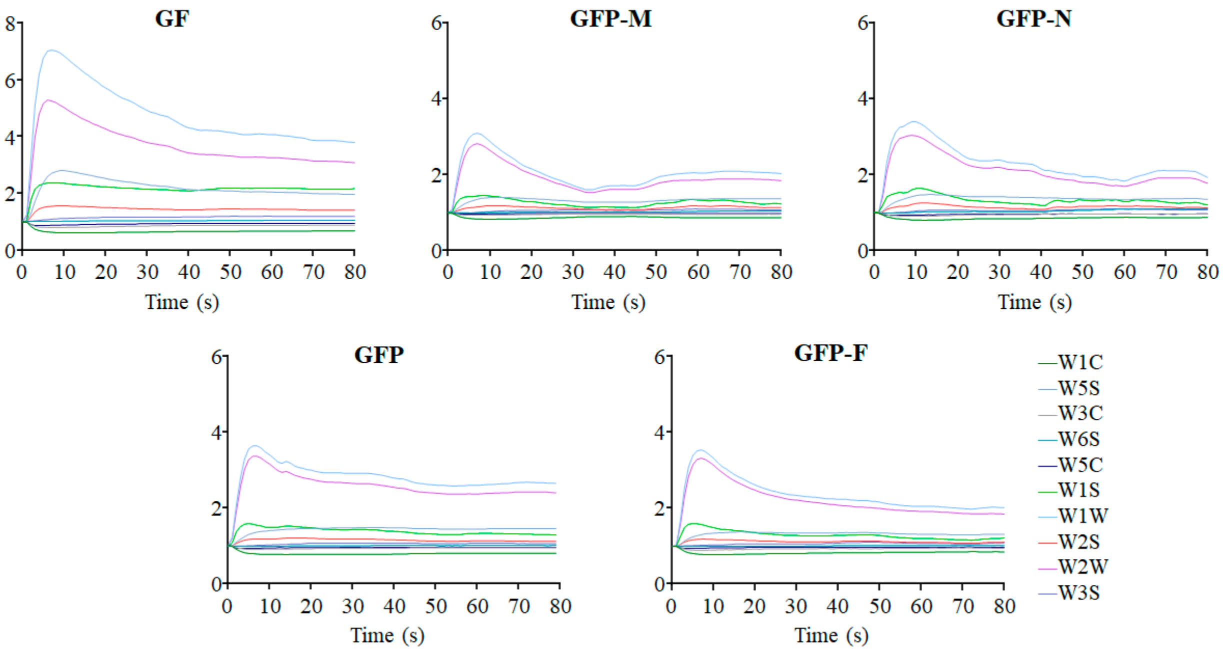

Figure S1. Response curve of sensors of electronic nose.

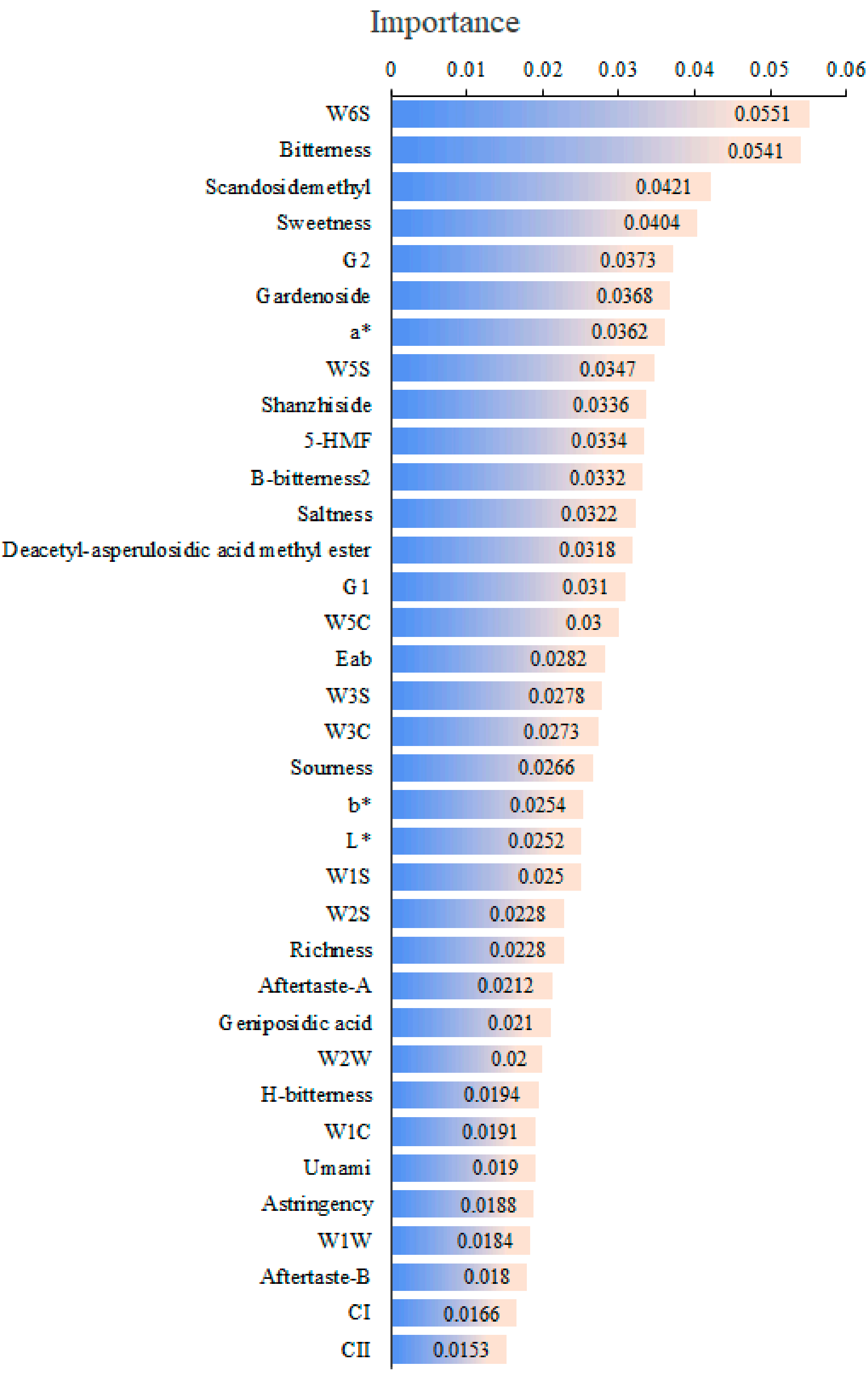

Figure S2. Variable importance of neural network model.

Classification for

Overall Percent Correct = 100.0%

Observed	Predicted				
	GF	GFP	GFP-F	GFP-M	GFP-N
GF	100.0%	0.0%	0.0%	0.0%	0.0%
GFP	0.0%	100.0%	0.0%	0.0%	0.0%
GFP-F	0.0%	0.0%	100.0%	0.0%	0.0%
GFP-M	0.0%	0.0%	0.0%	100.0%	0.0%
GFP-N	0.0%	0.0%	0.0%	0.0%	100.0%

Row Percent
100.00
80.00
60.00
40.00
20.00
0.00

Figure S3. Overall percentage correction of neural network model.
